# Optimal surgical methods to treat intertrochanteric fracture: a Bayesian network meta-analysis based on 36 randomized controlled trials

**DOI:** 10.1186/s13018-020-01943-9

**Published:** 2020-09-10

**Authors:** Yan-xiao Cheng, Xia Sheng

**Affiliations:** Department of Orthopedics, Jingjiang People’s Hospital, No.28, Zhongzhou Road, Jingjiang, Taizhou City, 214500 Jiangsu Province China

**Keywords:** Intertrochanteric fracture, Surgical interventions, Harris hip scores, Network meta-analysis

## Abstract

**Background:**

There are several surgical methods to treat intertrochanteric fracture: dynamic hip screw (DHS), compression hip screw (CHS), percutaneous compression plate (PCCP), Medoff sliding plate, less invasive stabilization system (LISS), Gamma nail, proximal femoral nail (PFN), and proximal femoral nail anti-rotating (PFNA). We therefore conducted a network meta-analysis to compare eight surgical interventions, including DHS, CHS, PCCP, Medoff sliding plate, LISS, Gamma nail, PFN, and PFNA, to provide the optimal surgical intervention for intertrochanteric fracture.

**Methods:**

An electronic search of 4 databases (PubMed, Embase, Cochrane library, and Web of Science) from inception to July 2020. Two or more of the eight surgical interventions, including the DHS, CHS, PCCP, Medoff sliding plate, LISS, Gamma nail, PFN, and PFNA, for intertrochanteric fracture were included. The methodological quality of the included studies was assessed using the Cochrane Collaboration risk of bias (ROB) tool. Network meta-analysis was conducted by using R-3.5.1 software with the help of package “gemtc”. The odd ratios (ORs) with 95% credibility interval (CrI) were used to assess complications and standard mean difference (SMD) with 95% CrI to calculate the continuous outcomes (operative time, intraoperative blood loss, and Harris hip score). Surfaces under the cumulative ranking curves (SUCRA) were used to rank the intervention.

**Results:**

A total of 36 RCTs were included in this study. The results of this network meta-analysis showed that, compared with the CHS and DHS group, PFNA exhibited a beneficial role in reducing the blood loss (SMD, 152.50; 95% CrI, 72.93 to 232.45; and SMD, 184.40; 95% CrI, 132.99 to 235.90, respectively). PFNA achieved the lowest value for the surface under the cumulative ranking curve (SUCRA) for the blood loss (SURCA = 0.072) and highest of Harris hip score (SURCA = 0.912). PCCP may have the lowest probability of the operative time (SURCA = 0.095). There were no significant differences among the eight surgical procedures in complications.

**Conclusion:**

PFNA technique is the optimal treatment method for intertrochanteric fracture. Larger, longitudinal RCTs addressing current limitations, including sources of bias, inconsistency, and imprecision, are needed to provide more robust and consistent evidence.

## Background

Intertrochanteric fractures are common injuries in elderly, with estimated prevalence of intertrochanteric fractures greater than 150,000 cases in the USA annually [1, 2]. Patients with intertrochanteric fractures always have a history of falls or bone disease, which might be due to a low-energy mechanism including fall from standing [3, 4]. Further, the typical clinical manifestations include pain and difficulty walking. The aging was associated with a greater risk of intertrochanteric fractures, and the mortality rate from intertrochanteric fractures ranged from 12 to 41% within 6 months [5]. The goal of treatment of patients with intertrochanteric fractures was to reduce the morbidity, mortality, re-operation, and early mobility [6].

Several surgical methods have already been demonstrated to be effective for patients with intertrochanteric fractures, mainly including extramedullary fixation (dynamic hip screw (DHS), compression hip screw (CHS), percutaneous compression plate (PCCP), Medoff sliding plate, and less invasive stabilization system (LISS)) and intramedullary fixation (Gamma nail, proximal femoral nail (PFN), and proximal femoral nail anti-rotating (PFNA)) [7–14]. Generally, intramedullary fixation is a valuable alternative method for patients with intertrochanteric fractures, which could be associated with lower levels of operation time, blood loss, and tissue damage.

The percutaneous compression plate (PCCP) was developed in the late 1990s by Gotfried for fixation in patients with intertrochanteric fractures [15]. This method could minimize operative trauma using two small percutaneous portals and small-diameter drilling, which could lower additional bone damage in the remaining lateral trochanteric wall. Previous studies illustrated that PFNA was associated with a lower risk of implant-related complications and could provide angular and rotational stability [16]. These characteristics, with early mobilization and weight-bearing, were suitable for patients with osteoporotic bone and unstable intertrochanteric fractures. LISS has some advantages in the treatment of complex proximal femoral fractures in a more stable construct with higher pullout resistance [17].

Previous studies have tested different internal fixation techniques for the surgical treatment to provide insight into the option for treating intertrochanteric fractures. However, there is no consensus about the optimal surgical method for intertrochanteric fractures [18, 19]. More important, traditional meta-analyses only compare two treatments, while network meta-analysis allows for the simultaneous comparison of multiple interventions through combination of direct and indirect evidences from RCTs.

Therefore, a Bayesian network meta-analysis was performed to compare eight common surgical methods, including DHS, CHS, PCCP, Medoff sliding plate, LISS, Gamma nail, PFN, and PFNA, to provide the optimum treatment method for intertrochanteric fracture.

## Material and methods

This systematic review was written according to the Preferred Reporting Items for Systematic Reviews and Meta-Analysis (PRISMA) Extension Statement for Meta-analysis. Ethical approval was not required as the work was collected data from published literatures.

### Search strategy

PubMed, Embase, Cochrane library, and Web of Science were searched for potentially relevant studies from the time of the inception to July 2020. The terms used for the literature search were as follows: “percutaneous compression plate” OR “proximal femoral nail anti-rotation” OR “proximal femoral nail” OR “less invasive stabilization system” OR “dynamic hip screw” OR “compression hip screw” OR “Medoff sliding plate” AND “intertrochanteric fractures”. In addition, we performed a manual search according to the references of eligible studies to prevent any omissions. Study topic, design, intervention, control, and investigated outcomes were employed to identify any included studies. The literature search and study selection were conducted by two authors independently using a standardized approach. Any inconsistency was resolved by group discussion until a consensus was reached.

### Inclusion and exclusion criteria

Studies were pooled for meta-analysis if they met the following criteria: (1) the study with RCTs; (2) the head to head RCT that compares any of the following two comparisons: DHS, CHS, PCCP, Medoff sliding plate, LISS, Gamma nail, PFN, and PFNA; (3) the study presenting the relevant outcomes, including blood loss, Harris hip score, operation time, and complications; and (4) intertrochanteric fractures were confirmed via X-ray imaging.

### Data extraction

Two reviewers (Yan-xiao Cheng and Xia Sheng) independently extracted data in pre-designed proforma and managed using Microsoft Excel 2010 (Microsoft Corp, Redmond, WA). Any discrepancy was resolved by a consensus meeting between the two reviewers. Following information including first author’s name, publication year, study design, sample size, mean age, percentage of males, Orthopaedic Trauma Association (OTA) fracture classification, and investigated outcomes (blood loss, Harris hip scores, operation time, and postoperative complications) were extracted.

### Quality assessment

The methodological quality of each randomized controlled trials (RCTs) was assessed according to the Cochrane Collaboration tool for assessing the risk of bias (ROB). A total of seven items were included for assessment: random sequence generation, allocation concealment, blinding of participant and personnel, blinding of outcome assessment, incomplete outcome data, selective outcome reporting, and other bias.

### Statistical analysis

Network meta-analysis was conducted using a Bayesian approach using R version 3.5.1 (R Project for Statistical Computing) through the library gemtc. Node splitting method will be used to evaluate the inconsistency between direct and indirect comparisons. There was no significant inconsistency when 95% CIs of inconsistency factors include zero or *P* value > 0.05 for the comparison between direct and indirect effects. Heterogeneity of study results was assessed using *I*^2^ test, and significant heterogeneity was considered at *I*^2^ > 50%. The clinical outcome (operation time, intraoperative blood loss, and Harris hip scores) was evaluated by the standard mean difference (SMD) with 95 % credibility interval (CrI). Postoperative complications were expressed as odds ratios (ORs) with 95% CIs. Comparison-adjusted funnel plots were performed by Stata 14.2 (Stata Corp, College Station, TX) to assess publication bias for network meta-analyses.

## Result

### Study characteristics

**Fig. 1:**
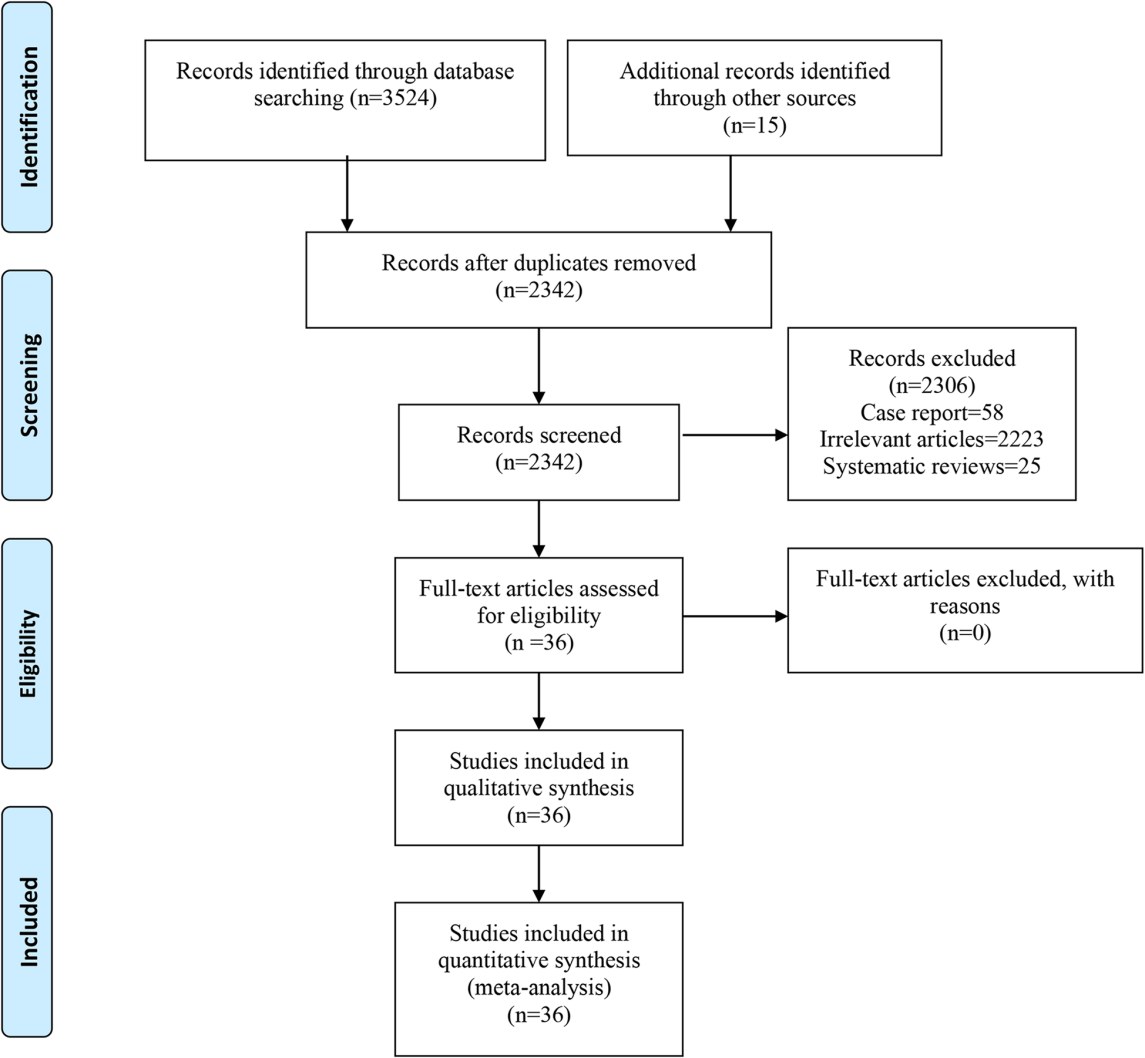
The flow diagram of study selection

A total of 3524 studies were identified from the electronic search, and additional 15 records were identified through other sources. Using Endnote software (Clarivate Analytics), a total of 1182 duplicated articles were excluded. A total of 2223 obviously irrelevant studies were excluded after reading the title and the abstract; another 83 studies were excluded due to various reasons after reading the full text. Finally, a total of 36 studies were included in this meta-analysis [20–52]. A flow chart diagram of the search strategy and study selection is provided in Fig. 1, and the general characteristics of the included studies are presented in Table 1.

**Table 1:** General characteristic of the included studies. *NA* not available, *RCT* randomized controlled trial, *DHS* dynamic hip screw, *CHS* compression hip screw, *PCCP* percutaneous compression plate, Medoff sliding plate, *LISS* less invasive stabilization system, *PFN* proximal femoral nail, *PFNA* proximal femoral nail anti-rotating

Authors	Intervention	Comparator	Follow-up (months)	Type of fracture	Age (mean, year)	Study	BMI (kg/m^2^)	Outcomes
Leung et al. [20]	Gamma nail	CHS	7.2	31-A1–A3	83.35	RCT	NA	1,2,3,4
Goldhagen et al. [21]	Gamma nail	DHS	6	31-A1–A3	80.6	RCT	NA	1,2,3,4
Butt et al. [22]	Gamma nail	DHS	6	31-A2–A3	79	RCT	NA	1,2,3,4
O’Brien et al. [23]	Gamma nail	DHS	13	31-A1–A2	81.2	RCT	NA	1,2,3,4
Hoffman and Lynskey [24]	Gamma nail	DHS	12	31-A1–A3	62.25	RCT	NA	1, 3,4
Kukla et al. [25]	Gamma nail	DHS	6	31-A1–A3	81.9	RCT	NA	1,2,3,4
Ahrengart et al. [26]	Gamma nail	CHS	6	31-A1–A3	79.6	RCT	NA	1,2,3,4
Kosygan et al. [27]	PCCP	CHS	19	31-A1–A3	72.95	RCT	NA	1,2,3,4
Utrilla et al. [28]	Gamma nail	CHS	12	31-A1–A3	80.2	RCT	NA	1,2,3,4
Ekström et al. [29]	Gamma nail	Medoff sliding plate	6	31-A2	83.2	RCT	NA	1,2,3,4
Peyser et al. [30]	PCCP	CHS	12	31-A2	80.85	RCT	NA	1,2,3,4
Romero et al. [31]	DHS	PCCP	12	31-A1–A3	82.9	RCT	NA	1,2,3,4
Zou et al. [32]	DHS	PFNA	12	31-A1–A3	82.5	RCT	NA	1,2,4
Xu et al. [33]	DHS	PFNA	12	31-A1–A3	62.25	RCT	NA	1,2,3,4
Yaozeng et al. [34]	Gamma nail	PFNA	6	31-A1–A3	NS	RCT	NA	1,3,4
Yang et al. [35]	PCCP	DHS	15	31-A1–A3	71.2	RCT	NA	1,2,3,4
Guo et al. [36]	PCCP	PFNA	12	31-A1–A2	83.55	RCT	NA	1,2,3,4
Sharma et al. [37]	PFNA	DHS	6	31-A1–A3	81	RCT	NA	1,2,3,4
Singh et al. [38]	PFNA	DHS	5	31-A1–A3	83.3	RCT	NA	1,2,3,4
Adeel et al. [39]	PFNA	DHS	12	31-A1–A3	82.5	RCT	NA	1,2,3,4
Brandt et al. [40]	PCCP	DHS	3	31-A1–A3	71.4	RCT	NA	1,2,3,4
Bridle et al. [41]	Gamma nail	DHS	6	31-A1–A3	NS	RCT	NA	1,2,3,4
Janzing et al. [42]	PCCP	DHS	12	31-A1–A3	76.2	RCT	NA	1,2,3,4
Kosygan et al. [27]	PCCP	DHS	19	31-A1–A3	83	RCT	23.2	1,2,3,4
Madsen et al. [43]	Gamma nail	DHS	6	31-A1–A3	82.9	RCT	NA	1,2,3,4
McCormack et al. [44]	DHS	Medoff sliding plate	6	31-A1–A3	81	RCT	NA	1,2,3
Miedel et al. [45]	Gamma nail	Medoff sliding plate	12	31-A1–A3	68.9	RCT	NA	1,2,3,4
O’Brien et al. [23]	Gamma nail	DHS	13	31-A1–A3	70.4	RCT	NA	1,2,3,4
Pajarinen et al. [46]	DHS	Gamma nail	4	31-A1–A2	80.2	RCT	21.8	1,2,3,4
Park et al. [47]	Gamma nail	CHS	12	31-A1–A3	76.2	RCT	NA	1,2,3,4
Parker et al. [48]	Gamma nail	CHS	12	31-A1–A2	72.9	RCT	NA	1,2,3,4
Radford et al. [49]	DHS	Gamma nail	12	31-A1–A3	NS	RCT	NA	1,2,3,4
Schipper et al. [50]	Gamma nail	PFN	12	31-A1–A3	NS	RCT	NA	1,3,4
Utrilla et al. [28]	Gamma nail	CHS	12	31-A2	NS	RCT	NA	1,2,3,4
Vaquero et al. [51]	PFNA	Gamma nail	12	31-A1–A3	72.85	RCT	NA	1,2,3,4
Zhou et al. [52]	LISS	PFNA	12	31-A1–A3	75.7	RCT	NA	1,2,3,4

**Fig. 2:**
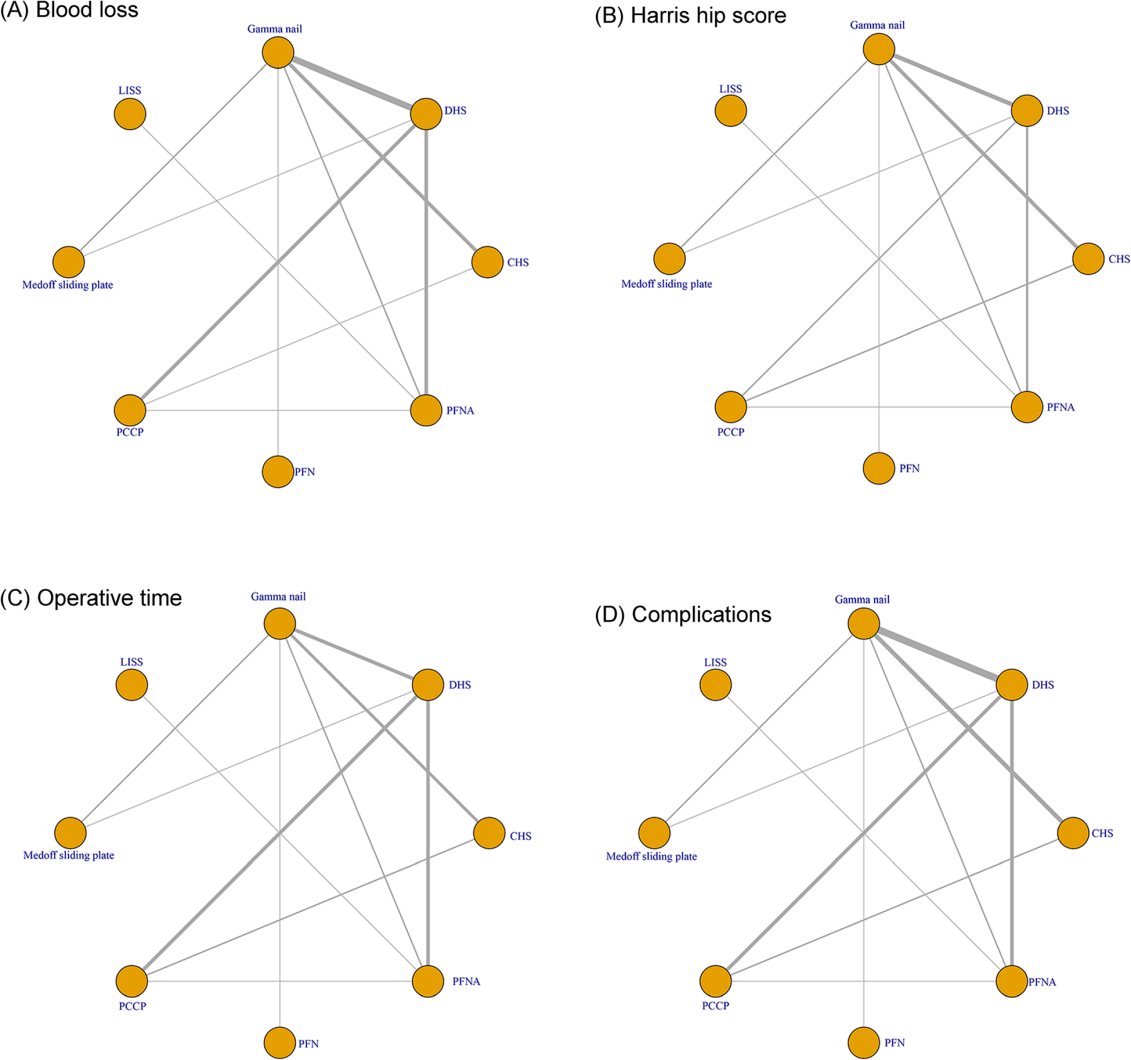
Evidence network of eligible comparisons for network meta-analysis. Numbers by the lines indicate the cumulative number of enrolled studies for each direct comparison

Six comparisons evaluated the effect of Gamma nail and CHS, 10 comparisons evaluated the effect of Gamma nail and DHS, 5 comparisons evaluated the effect of PFNA and DHS, 2 comparisons evaluated the PCCP and CHS, 2 comparisons evaluated the effect of PCCP and CHS, 2 comparisons evaluated the Gamma nail and PFNA, 1 comparison evaluated the Gamma nail and PFN, 1 study evaluated LISS and PFNA, 1 study evaluated DHS and Medoff sliding plate, 1 study evaluated PCCP and PFNA, and 1 study evaluated PCCP and DHS. Follow-up duration ranged from 3 to 19 months (mean follow-up duration = 10.06 months). Figure 2 graphically represents the network of eligible comparisons for the blood loss, Harris hip score, operative time, and complications of the network meta-analysis.

### Methodological quality

**Fig. 3:**
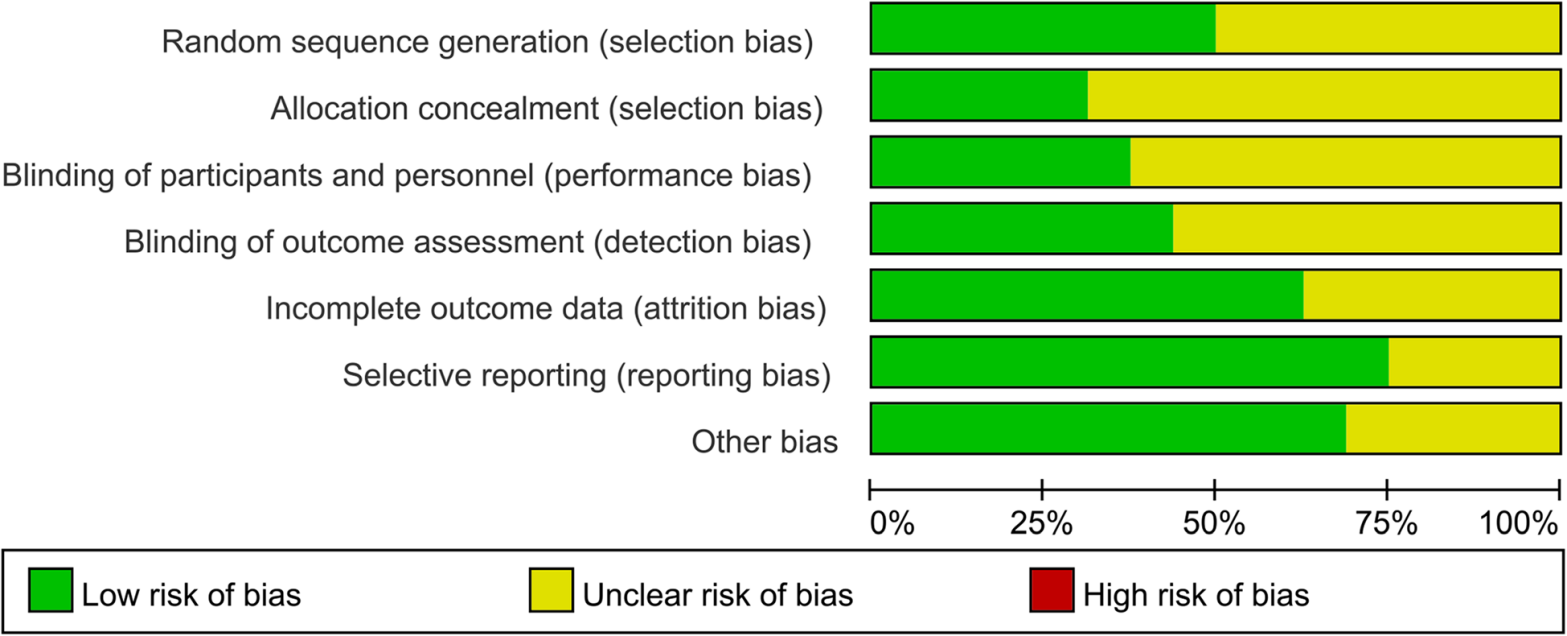
Risk of graph of the included studies

All included studies in the meta-analysis were judged to be at high/unclear risk of bias. High/unclear risk of bias was assessed because all included studies had not described adequate blind method and sample calculation. Random sequence generation was adequate in only 12 studies. The details regarding the risk of bias for each included study are shown in Fig. 3.

### Results from network meta-analysis

#### Blood loss

A total of 35 studies reported the intraoperative blood loss. Pooled results revealed that Gamma nail could significantly decrease blood loss than CHS (SMD, 76.16; 95% CrI, 17.78 to 134.71, Table 2) and DHS (SMD, 108.05; 95% CrI, 67.16 to 149.07, Table 2). Moreover, Gamma nail could decrease blood loss than LISS (SMD, − 197.87; 95% CrI, − 349.78 to − 45.44, Table 2) and Medoff sliding plate (SMD, − 150.74; 95% CrI, − 232.93 to − 68.17, Table 2).

**Table 2:**
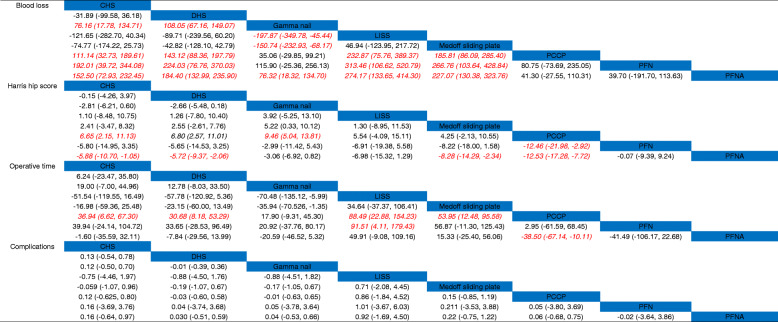
The comparison of eight surgical methods for blood loss, Harris hip score, operative time and complications according to the network meta-analysis using standard mean difference or odds ratios (ORs) and corresponding 95 % credential intervals (Crls). Italics with red colors were with statistically significant

PCCP could significantly decrease the blood loss than CHS (SMD, 111.14; 95% CrI, 32.73 to 189.61, Table 2), DHS (SMD, 143.12; 95% CrI, 88.36 to 197.79, Table 2), LISS (SMD, 232.87; 95% CrI, 75.76 to 389.37), and Medoff sliding plate (SMD, 185.81; 95% CrI, 86.09 to 285.40, Table 2).

PFN could also decrease blood loss than CHS (SMD, 192.01; 95% CrI, 39.72 to 344.08, Table 2), DHS (SMD, 224.03; 95% CrI, 76.76 to 370.03, Table 2), LISS (SMD, 313.46; 95% CrI, 106.62 to 520.79, Table 2), and Medoff sliding plate (SMD, 266.76; 95% CrI, 103.64 to 428.84, Table 2).

The results of this network meta-analysis showed that, compared with the CHS and DHS group, PFNA exhibited a beneficial role in reducing the blood loss (SMD, 152.50; 95% CrI, 72.93 to 232.45; and SMD, 184.40; 95% CrI, 132.99 to 235.90, respectively, Table 2). Compared with Gamma nail, PFNA was associated with a reduction of the blood loss (SMD, 76.32; 95% CrI, 18.32 to 134.70, Table 2).

#### Harris hip score

Thirty studies were available to assess the effect of eight treatment methods on postoperative Harris hip score. We observed that CHS has a higher Harris hip score than PCCP (SMD = 6.65, 95 % CrI 2.15–11.13, Table 2). Gamma nail could also increase the Harris hip score than PCCP (SMD = 9.46, 95 % CrI 5.04–13.81, Table 2).

The results of this network meta-analysis showed that, compared with the CHS, DHS, Medoff sliding plate, and PCCP group, PFNA exhibited a beneficial role in increasing the Harris hip score (SMD, − 5.88; 95% CrI, − 10.70, − 1.05; SMD, − 5.72; 95% CrI, − 9.37 to − 2.06; SMD, − 8.28; 95% CrI, − 14.29 to − 2.34; SMD, − 12.53; 95% CrI, − 17.28 to − 7.72, respectively, Table 2).

#### Operative time

Twenty-eight studies reported different treatment methods for the operative time. We found that PCCP could significantly decrease the operative time than CHS (SMD, 36.94, 95% CrI, 6.62 to 67.30, Table 2), DHS (SMD, 30.68; 95% CrI, 8.18 to 53.29, Table 2), LISS (SMD, 88.49; 95% CrI, 22.88 to 154.23, Table 2), and Medoff sliding plate (SMD, 53.29; 95% CrI,12.48 to 95.58, Table 2). Moreover, PCCP could significantly decrease the operative time than that of PFNA group (SMD, − 38.50; 95% CrI, − 67.14 to − 10.11, Table 2).

#### Complications

Thirty-two studies were available to assess the eight surgical treatments for postoperative complications. There was no statistical significance among these groups for complications (Table 2).

### Relative ranking of eight treatment methods

**Fig. 4:**
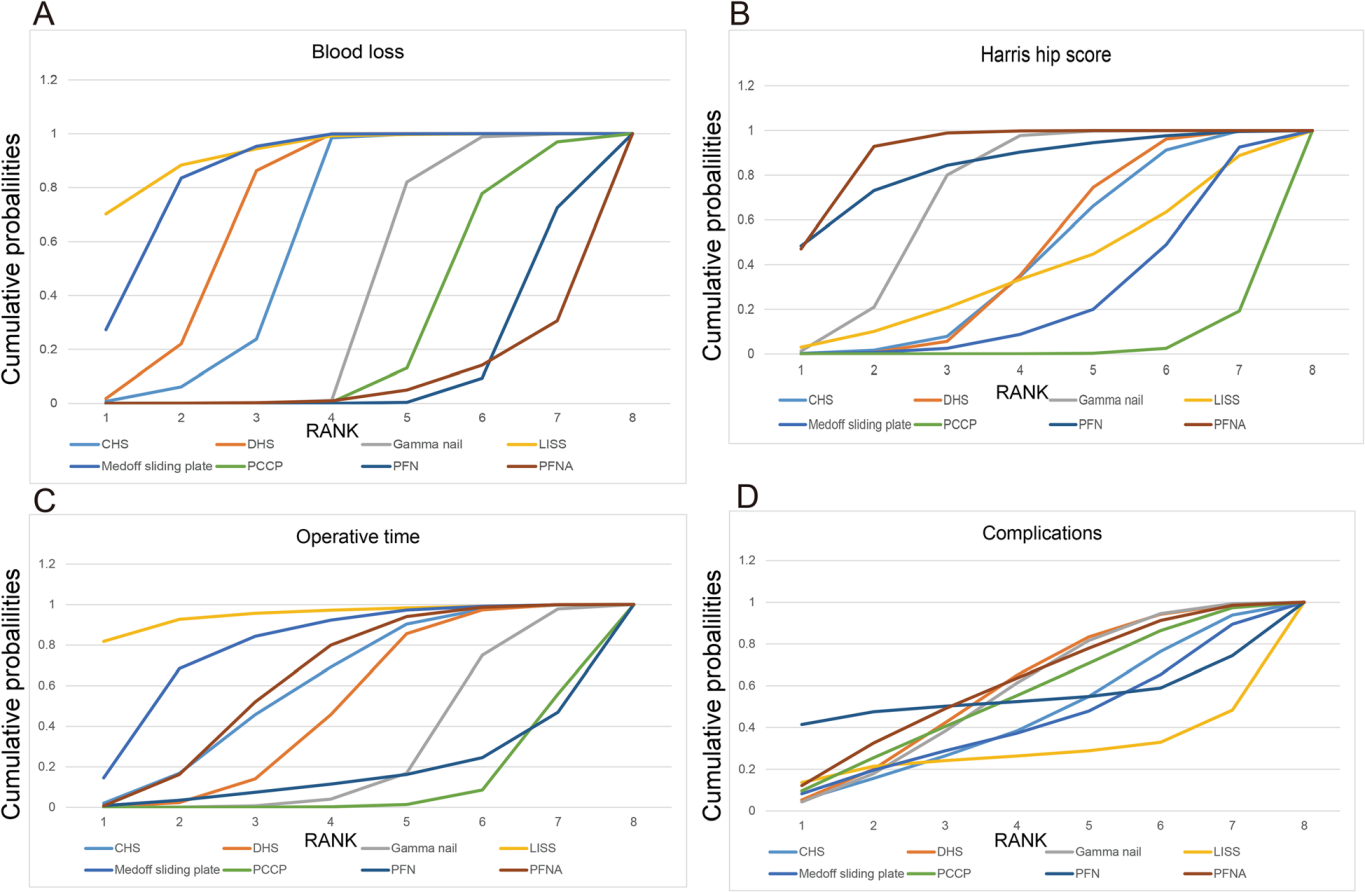
Surfaces under the cumulative ranking curves (SUCRA) for blood loss, Harris hip score, operative time and complications. The graph displays the distribution of probabilities for each treatment. The *X*-axis represents the rank, and the *Y*-axis represents probabilities

Figure 4a reveals the SUCRA probability of the blood loss for the eight surgical methods. PFNA may have the lowest probability of the blood loss (SURCA = 0.072). In Fig. 4b, we summarized the SUCRA probability of the Harris hip score for the eight treatment methods. PFNA may have the highest probability of the Harris hip score (SURCA = 0.912).

Figure 4c summarizes the SUCRA probability of the eight surgical methods for operative time. PCCP may have the lowest probability of the operative time (SURCA = 0.095). We observed that LISS may have the lowest probability of the incidence of complications (SURCA = 0.280, Fig. 4d).

### Comparisons between direct and indirect evidences

**Fig. 5:**
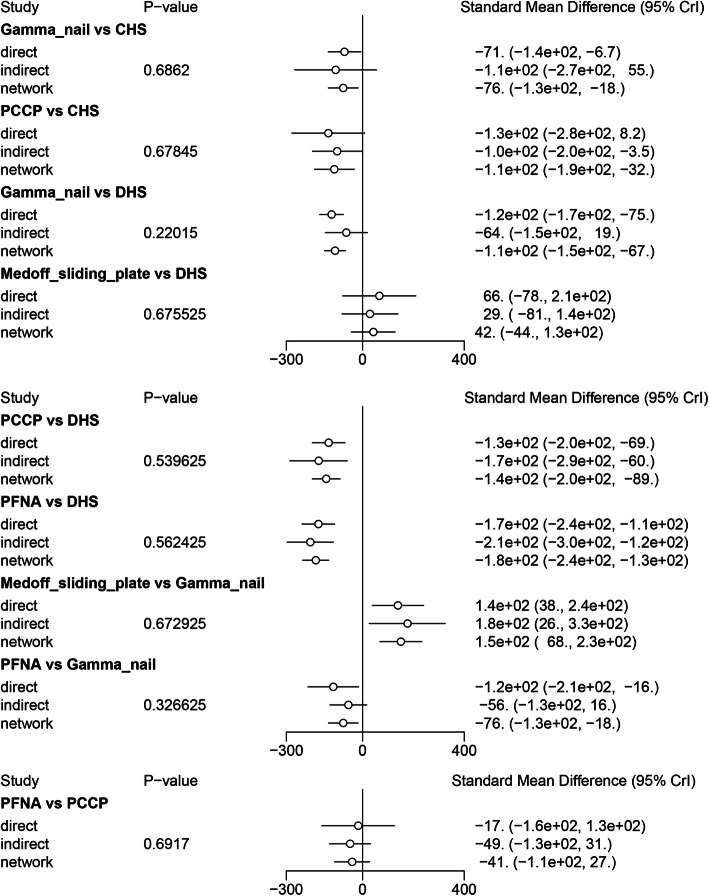
Comparison between direct and indirect evidence—blood loss

**Fig. 6:**
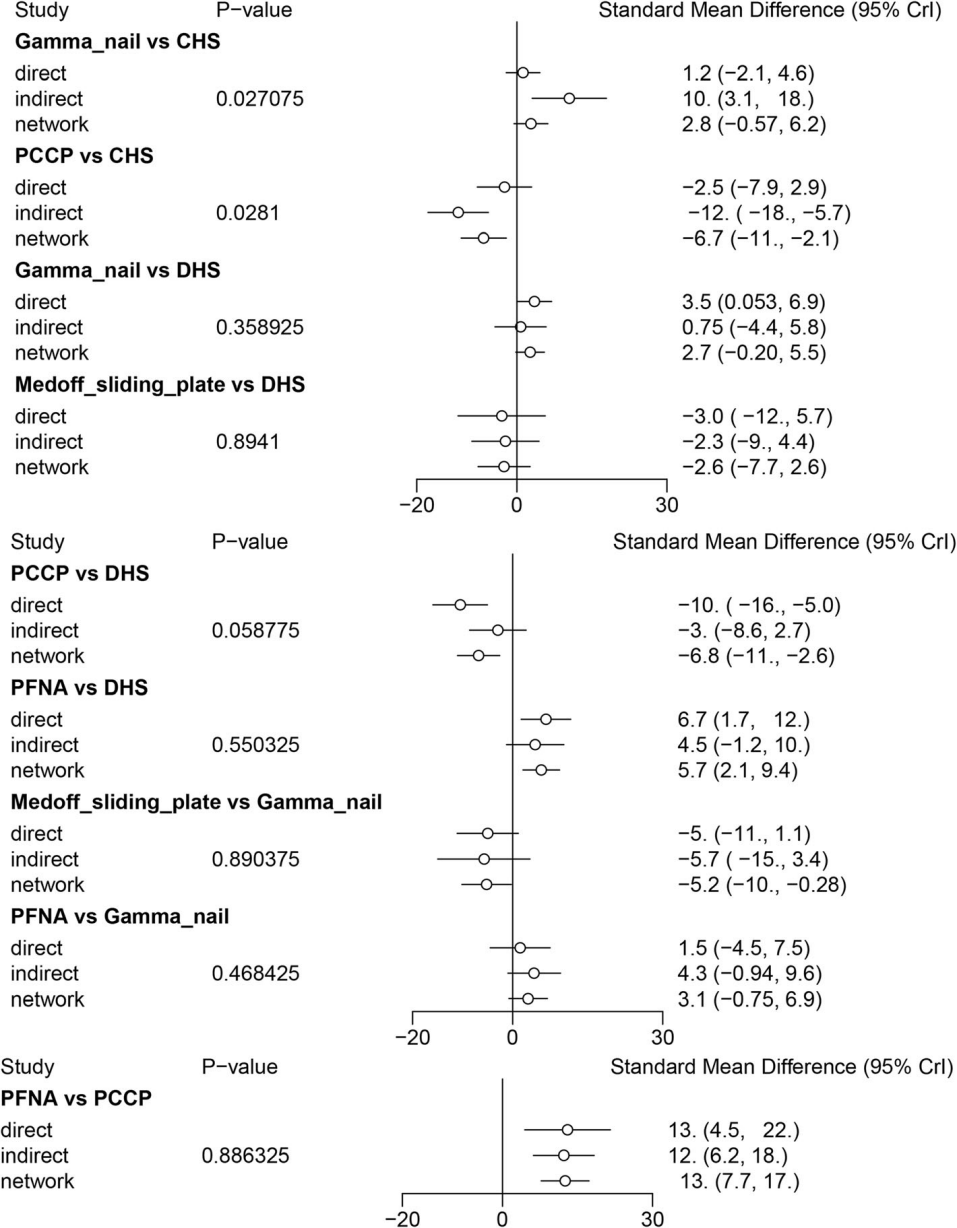
Comparison between direct and indirect evidence—Harris hip score

**Fig. 7:**
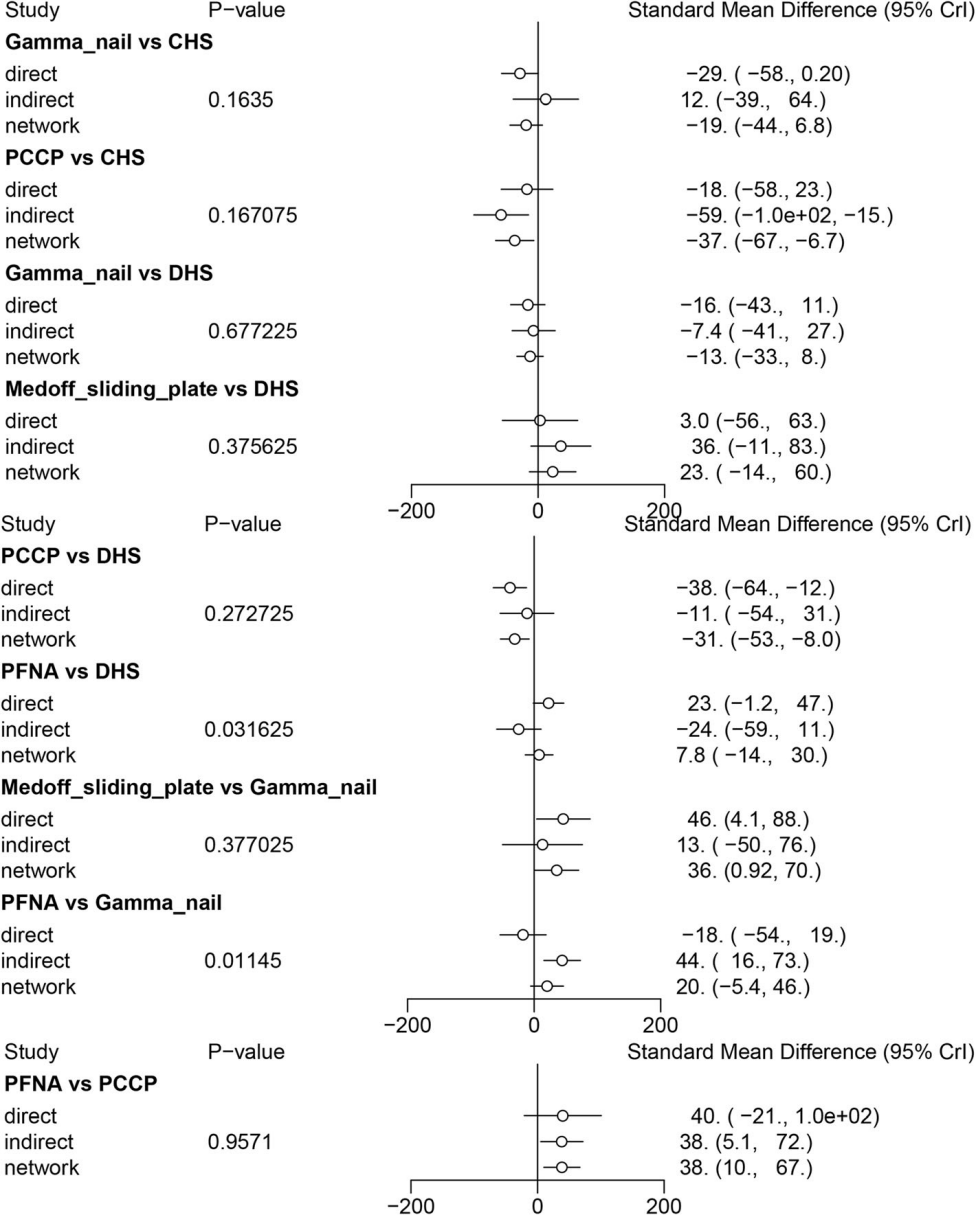
Comparison between direct and indirect evidence—operative time

**Fig. 8:**
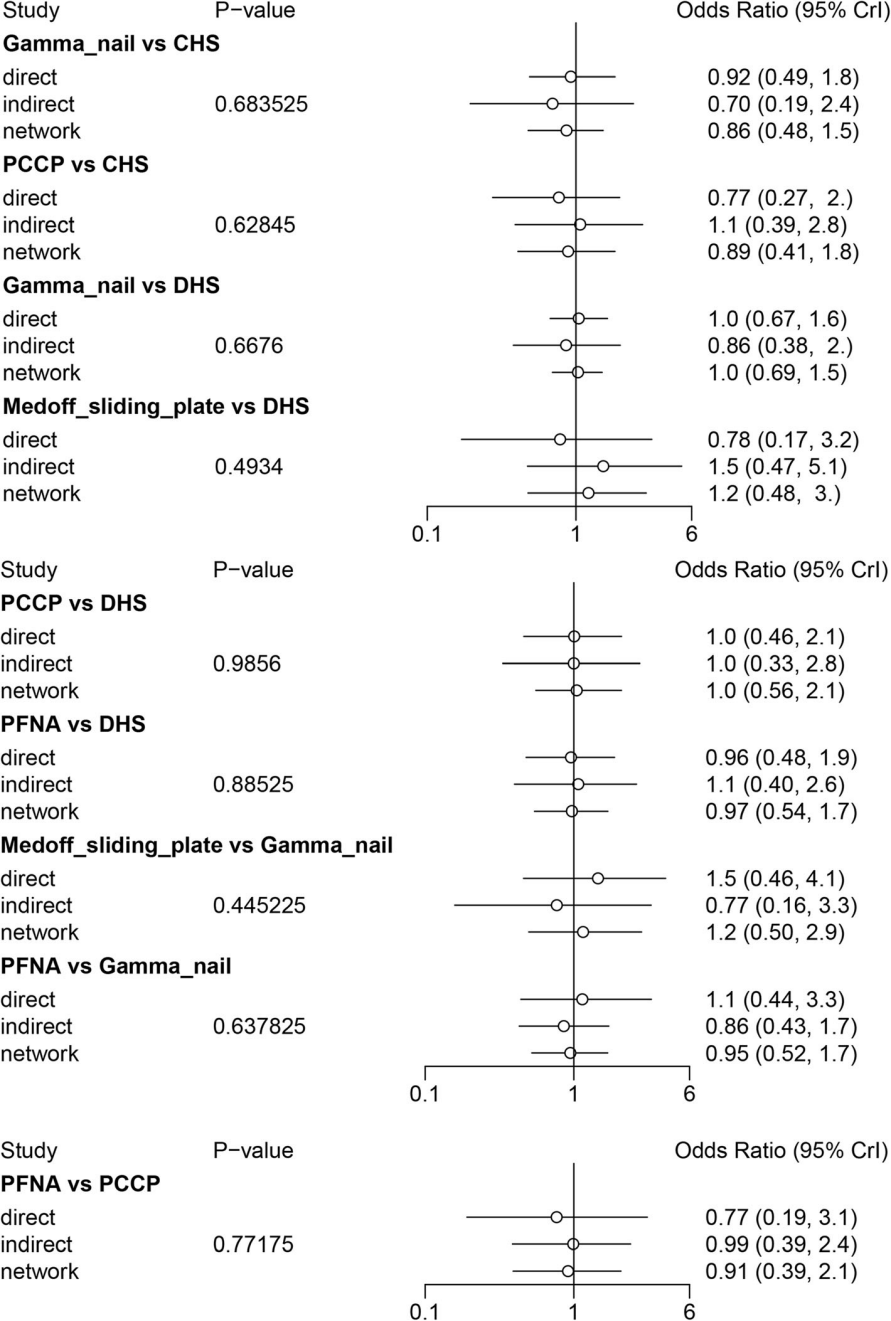
Comparison between direct and indirect evidence—complications

The inconsistency between direct and the indirect estimates for each comparison will be further confirmed by node-splitting method. Bayesian *P* value more than 0.05 indicated that there was no inconsistency of our results. We could easily find that all the *P* values of node-splitting method were above 0.05, which indicated the consistency of the direct and indirect evidence for blood loss (Fig. 5). However, significant differences were observed at the comparison between Gamma nail versus CHS and PCCP versus CHS for Harris hip score (Fig. 6). Other comparisons were all above 0.05, which indicated the consistency of the direct and indirect evidence for Harris hip score. As for operative time, *P* values of node-splitting method were above 0.05, except for PFNA versus DHS and PFNA versus Gamma nail (*P* < 0.05, Fig. 7). Nevertheless, no significant difference between direct and indirect evidence was observed in complications (Fig. 8).

## Discussion

In this network meta-analysis based on 36 RCTs, we systematically reviewed the DHS, CHS, PCCP, MSP, LISS, Gamma nail, PFN, and PFNA for treatment of intertrochanteric fracture. Thirty-six eligible studies were finally involved in this network meta-analysis. PFNA ranked as the most preferable surgical method with less blood loss and higher Harris hip score. As for operative time, PCCP may have the lowest probability of the operative time. However, complications did not differ among these groups. These results may help orthopedic surgeons for the selection of surgical methods for intertrochanteric fracture.

This is the very largest network meta-analysis that compared the efficacy and safety of eight common surgical methods for treatment of intertrochanteric fracture. Previously, Jiang et al. [53] conducted a meta-analysis about efficacy and safety of PFNA and LISS for intertrochanteric fracture; results suggested that PFNA could significantly reduce the hospital stay than LISS. This result is inconsistent with other observations. Arirachakaran et al. [54] suggested that PCCP was superior than DHS and PFNA in terms of intraoperative outcomes and postoperative complications. However, there were some limitations, including the retrospective study design, mixed PFN and PFNA into a group, and omitted important indicators for hip function. PFNA possesses biological advantage, minimally invasive approach, and easy manipulation. We firstly used blood loss to assess intraoperative advantage between these eight surgical methods. SURCA rank suggested that the blood loss in PFNA ranked the lowest. A major limitation of this outcome is that there is lack of hidden blood loss in these surgical treatments. It needs to be emphasized that hidden blood loss in the operation cannot be overlooked [55, 56]. Therefore, it is urgent to verify the hidden blood loss in these surgical interventions in future studies. Singh et al. [38] conducted a prospective randomized study and found that PFNA requires shorter surgical time and less blood loss than DHS.

For hip functions, we compared Harris hip scores as the main outcome. Regarding the increase of the Harris hip score, PFNA treatment was also ranked as the top intervention. These results suggested that PFNA could enhance the recovery of the hip function. Xie et al. [57] conducted a controlled study and suggested that PFNA had a better hip recovery than hemi-arthroplasty in intertrochanteric fractures. Ma et al. [58] conducted a meta-analysis about Gamma nail, PFNA, and DHS for intertrochanteric fracture. Results have shown that PFNA was a priority choice with minimal rate of fixation failure and shorter length of hospital stay.

We also compared operative time among the eight surgical methods. Results suggested that PCCP had the lowest probability of operative time than other treatments. However, other studies have drawn the opposite conclusion [19]. Hao et al. [19] suggested that PFNA treatment results in shortest operative time than other surgical treatments. As surgical experience might influence the operative time, thus more validation studies need to be performed. We finally compared complications between these eight treatments; network meta-analysis found that these eight treatments have no statistical significance. Concerning clinical safety, all of these treatments were comparable.

This network meta-analysis had several limitations. The number of included studies was limited and the sample size was small. Further, the quality of this network meta-analysis is limited by the quality of available literatures (high/unclear risk of bias). Additionally, the length of load time and postoperative rehabilitation strategies might be further affected by the patients’ hip functions. Finally, inconsistency was observed in places (Harris hip score and operative time); further research will be needed to verify it.

## Conclusion

PFNA technique is the optimal treatment method for intertrochanteric fracture. Larger, longitudinal RCTs addressing current limitations, including sources of bias, inconsistency, and imprecision, are needed to provide more robust and consistent evidence.

## Data Availability

We state that the data will not be shared since all the raw data are present in the figures included in the article.

## Abbreviations

DHS: Dynamic hip screw
CHS: Compression hip screw
PCCP: Percutaneous compression plate
LISS: Less invasive stabilization system
PFN: Proximal femoral nail
PFNA: Proximal femoral nail anti-rotating
RCTs: Randomized controlled trials
ORs: Odd ratios
CrI: Credibility interval
SMD: Standard mean difference
SUCRA: Surfaces under the cumulative ranking curves
OTA: Orthopaedic Trauma Association
ROB: Risk of bias
